# Mucoepidermoid carcinoma of the breast: A case report and literature review focused on radiological findings

**DOI:** 10.1097/MD.0000000000029745

**Published:** 2022-06-30

**Authors:** Seongjun Bak, Hye Young Choi, Jeong-Hee Lee, Jae Beom Na, Dae Seob Choi, Jae Min Cho, Ho Cheol Choi, Mi Jung Park, Ji Eun Kim, Hwa Seon Shin, Jung Ho Won, Ju-Yeon Kim, Jae-Myung Kim

**Affiliations:** a Department of Radiology, College of Medicine, Gyeongsang National University and Gyeongsang National University Hospital, Jinju, Republic of Korea; b Department of Pathology, Gyeongsang National University College of Medicine and Gyeongsang National University Hospital, Jinju, Republic of Korea; c Department of Surgery, Gyeongsang National University College of Medicine and Gyeongsang National University Hospital, Jinju, Republic of Korea.

**Keywords:** breast, mammography, MRI, mucoepidermoid carcinoma, ultrasonography

## Abstract

Mucoepidermoid carcinoma (MEC) of the breast is a rare entity, with an estimated incidence of only 0.2% to 0.3% of all primary breast tumors. The radiological features of breast MEC have scarcely been investigated mainly because of its rarity. In this article, we present a case of breast MEC diagnosed at our hospital and review the literature, focusing on radiological findings and radiologic–pathologic correlations that could improve clinical management of this entity. To the best of our knowledge, our study is the first review of the literature that focuses on the radiological features of breast MEC.

**Patient concerns:**

A 47-year-old premenopausal woman presented with a painless palpable mass in the right breast.

**Diagnosis:**

Mammography and ultrasonography revealed a mass with suspicious malignant features, which was categorized as Breast Imaging Reporting and Data System category 4c. A 14-gauge core-needle biopsy revealed an intermediate-grade MEC of the breast. The patient underwent breast magnetic resonance imaging and chest computed tomography for preoperative evaluation. Postoperative histopathological examination confirmed a diagnosis of intermediate-grade MEC. The clinical staging was T2N0M0.

**Interventions:**

The patient underwent breast-conserving surgery, adjuvant chemotherapy, radiotherapy, and hormonal therapy.

**Outcomes:**

No evidence of recurrence has been reported over 37 months.

**Lessons:**

The imaging characteristics of breast MEC were variable, and there were no specific radiological features for diagnosis. The presence of cystic components on radiological imaging is likely to be an indicator of a low-grade tumor and better prognosis, although the number of reported cases is limited.

## 1. Introduction

Mucoepidermoid carcinoma (MEC) is a tumor that arises most commonly in the salivary glands.^[1]^ MEC of the breast is a rare entity, with an estimated incidence of only 0.2% to 0.3% of all primary breast tumors.^[2]^ Only 43 cases of breast MEC have been reported in the literature to date,^[3,4]^ and the clinicopathological features of breast MEC have not yet been fully identified. Moreover, most previous studies were reported from a pathological perspective because the diagnosis of breast MEC depends on the pathological examination. Therefore, radiological features of breast MEC have rarely been investigated, with only a few previously published articles describing the radiological findings.^[3–10]^

In this study, we present a case of breast MEC diagnosed at our institution and review the literature focused on radiological findings and radiologic–pathologic correlations that could better understand this entity and potentially improve clinical practice. To our knowledge, our study is the first case of breast MEC reported in Korea and the first review of the literature focusing on the radiological features of breast MEC.

## 2. Case report

**Figure 1.**
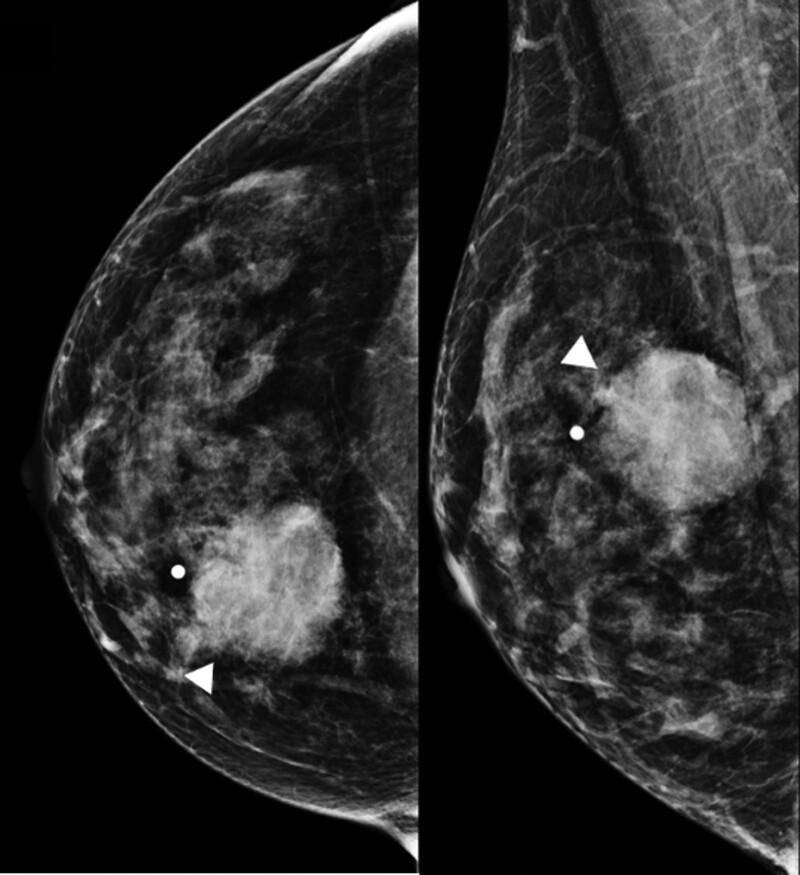
Mammography showing a 3.5 × 3.1 cm irregular, microlobulated, and hyperdense mass (arrowheads) in the upper inner quadrant of the right breast, and microcalcifications are not observed in the mass.

**Figure 2.**
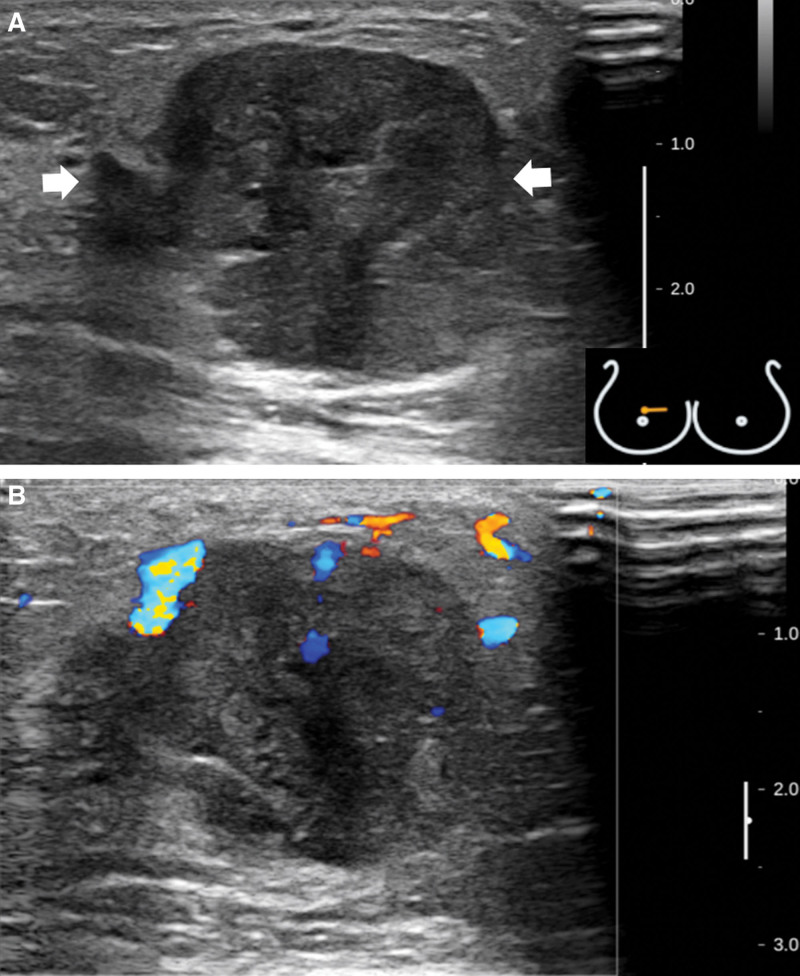
(A) A 3.4 × 2.3 cm irregularand heterogeneous echoic mass (arrows) with posterior acoustic enhancement at 2 o’clock of the right breast is revealed via ultrasonography. (B) Internal vascularity identified in the mass on color Doppler imaging. The lesion is categorized as Breast Imaging Reporting and Data System category 4c.

**Figure 3.**
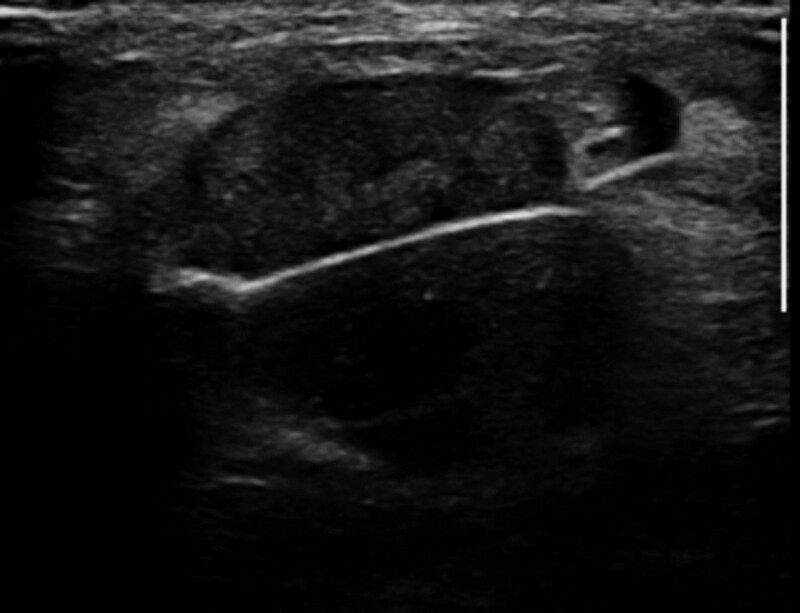
Ultrasound-guided core-needle biopsy revealed MEC of the breast, intermediate grade. MEC = mucoepidermoid carcinoma.

**Figure 4.**
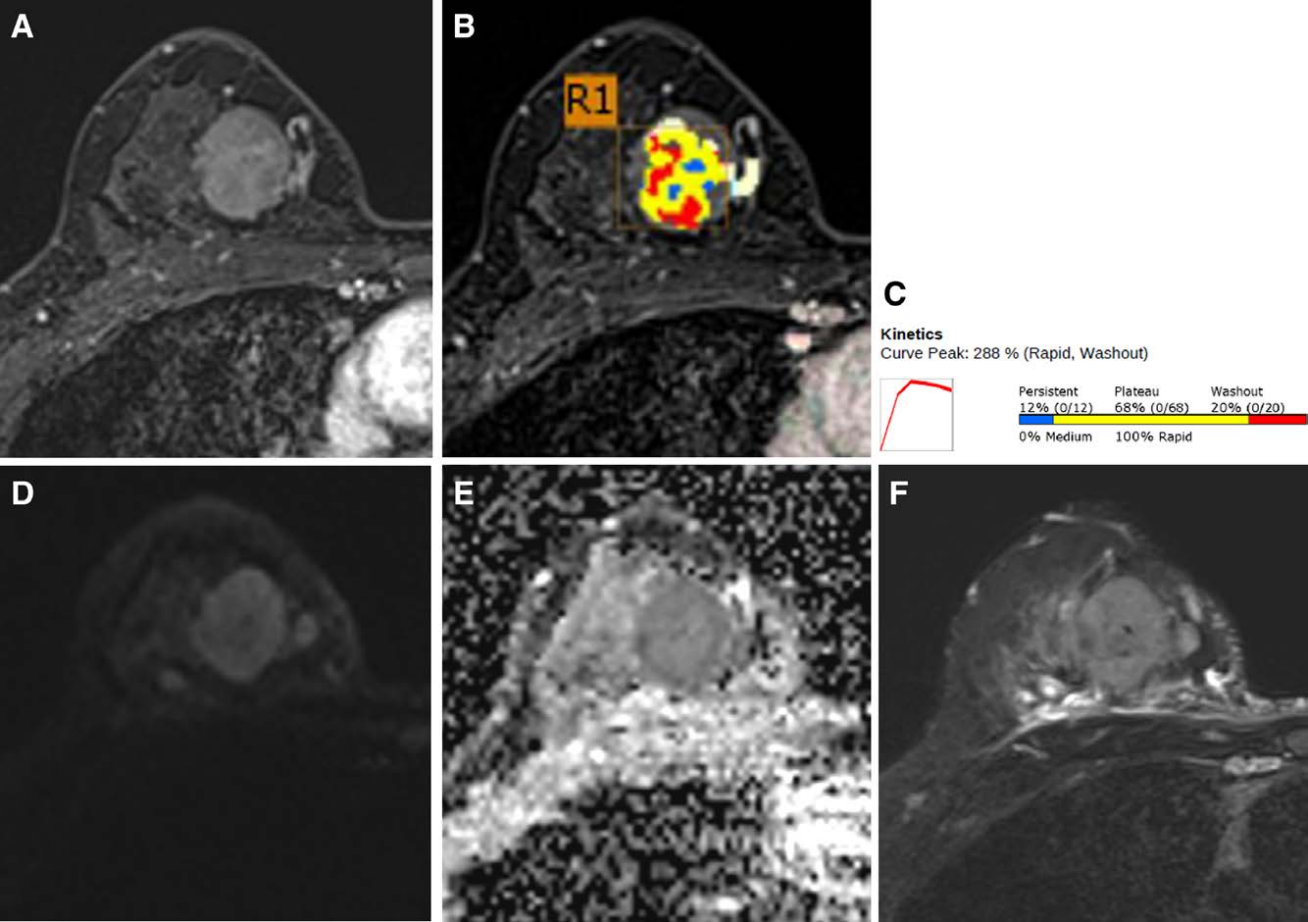
(A) Preoperative axial fat-suppressed T1-weighted contrast-enhanced MR image showing a 3.4 × 2.7 × 3.0 cm irregular, heterogeneously enhancing mass in the upper inner quadrant of the right breast. (B) MR image with CAD color overlay map demonstrating tumor enhancement kinetics. Red, yellow, and blue areas indicate washout, plateau, and persistent enhancement patterns, respectively. (C) Kinetic curve graph showing rapid initial enhancement and rapid washout-type curve. The initial peak enhancement value is 288%. In the delayed phase, 20% of the mass shows washout, 68% of the mass shows a plateau-type, and 12% shows a persistent-type enhancement. (D) T2-weighted image showing no cystic component in the mass. (E and F) Diffusion-weighted image (*b* = 1000) and ADC map show no significant diffusion restriction. ADC = apparent diffusion coefficient; CAD = computer-aided detection, MR = magnetic resonance.

**Figure 5.**
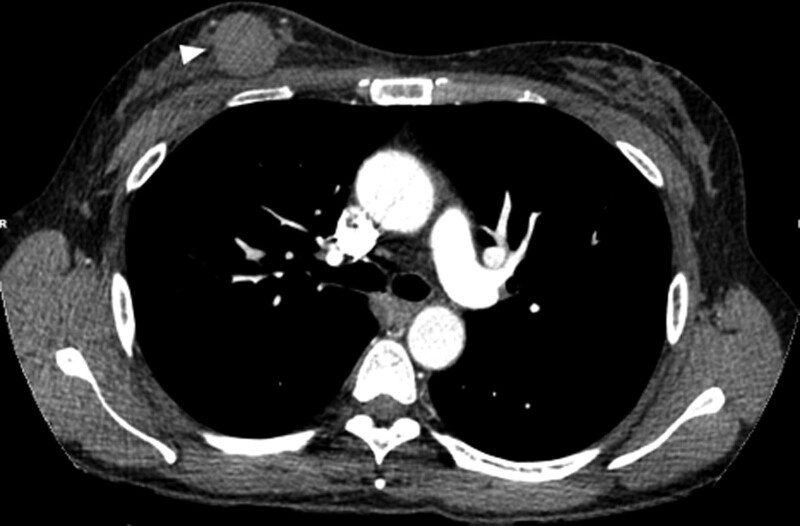
On preoperative chest CT scan, a 3.0 × 2.8 cm relatively well-defined homogeneously enhancing mass (arrowhead) is detected in the right breast. CT = computed tomography.

A 47-year-old premenopausal woman was referred to us for evaluation of a painless palpable mass discovered 7 months ago in the right breast. The patient had no remarkable medical or family history of breast cancer. On physical examination, a 3-cm lump was palpated in the upper inner quadrant of the right breast. No skin changes or nipple discharge were observed. The axillary lymph nodes were not palpable. Mammography (MG) showed an irregular, microlobulated, and hyperdense mass measuring 3.5 × 3.1 cm in the upper inner quadrant of the right breast (Fig. 1). Microcalcifications were not observed in the mass. Ultrasonography (US) with a 15-4-MHz high-frequency linear probe revealed a 3.4 × 2.3 cm irregular and heterogeneous echoic mass with posterior acoustic enhancement at 2 o’clock of the right breast (Fig. 2A), which was correlated with the mammographic mass. Internal vascularity was identified in the mass on color Doppler imaging (Fig. 2B). The lesion was categorized as Breast Imaging Reporting and Data System (BI-RADS) category 4c. No significantly enlarged axillary lymph nodes were observed. Ultrasound-guided 14-gauge core-needle biopsy was performed (Fig. 3) and revealed intermediate-grade MEC of the breast. For preoperative evaluation, the patient underwent breast magnetic resonance imaging (MRI) and chest computed tomography scans. Dynamic contrast-enhanced T1-weighted MRI images demonstrated an irregular, heterogeneously enhancing mass measuring 3.4 × 2.7 × 3.0 cm in the upper inner quadrant of the right breast (Fig. 4A). MRI with computer-aided detection color overlay map showed all 3 types of enhancement patterns, washout, plateau, and persistent, demonstrating the heterogeneity of tumor enhancement kinetics (Fig. 4B). The kinetic curve graph showed the rapid initial enhancement and rapid washout-type curve (Fig. 4C). With regard to delayed phase enhancement, 20% of the mass showed washout, 68% of the mass showed a plateau-type, and 12% showed a persistent-type enhancement. T2-weighted imaging showed no cystic component in the mass and diffusion-weighted imaging showed no significant diffusion restriction (Fig. 4D−F). On the contrast-enhanced chest computed tomography scan, a 3.0 × 2.8 cm, relatively well-defined, homogeneously enhancing mass was detected in the right breast (Fig. 5).

**Figure 6.**
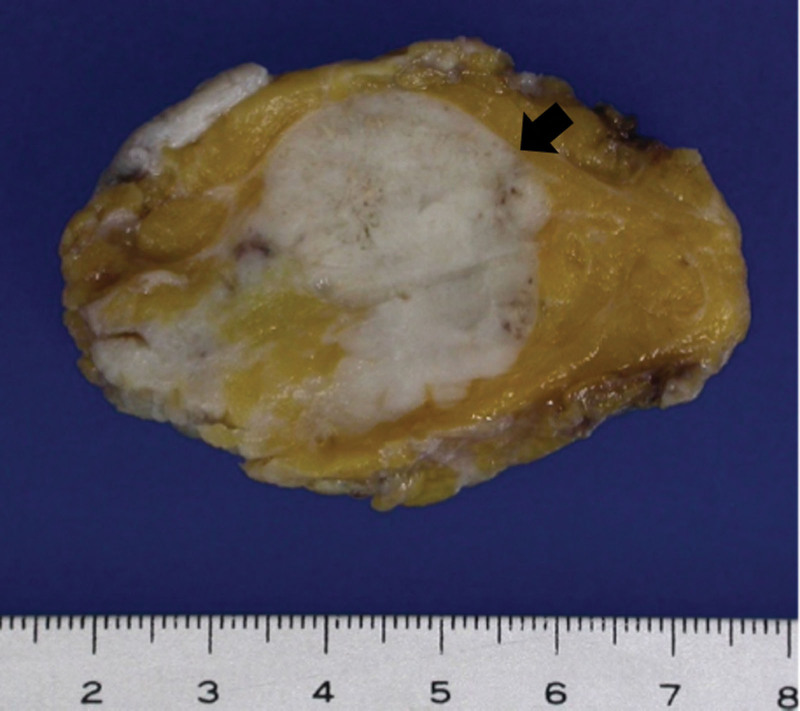
The cut surface of the breast-conserving surgery specimen showing a relatively well-circumscribed, whitish, and predominantly solid mass (arrows) with scattered tiny cysts.

**Figure 7.**
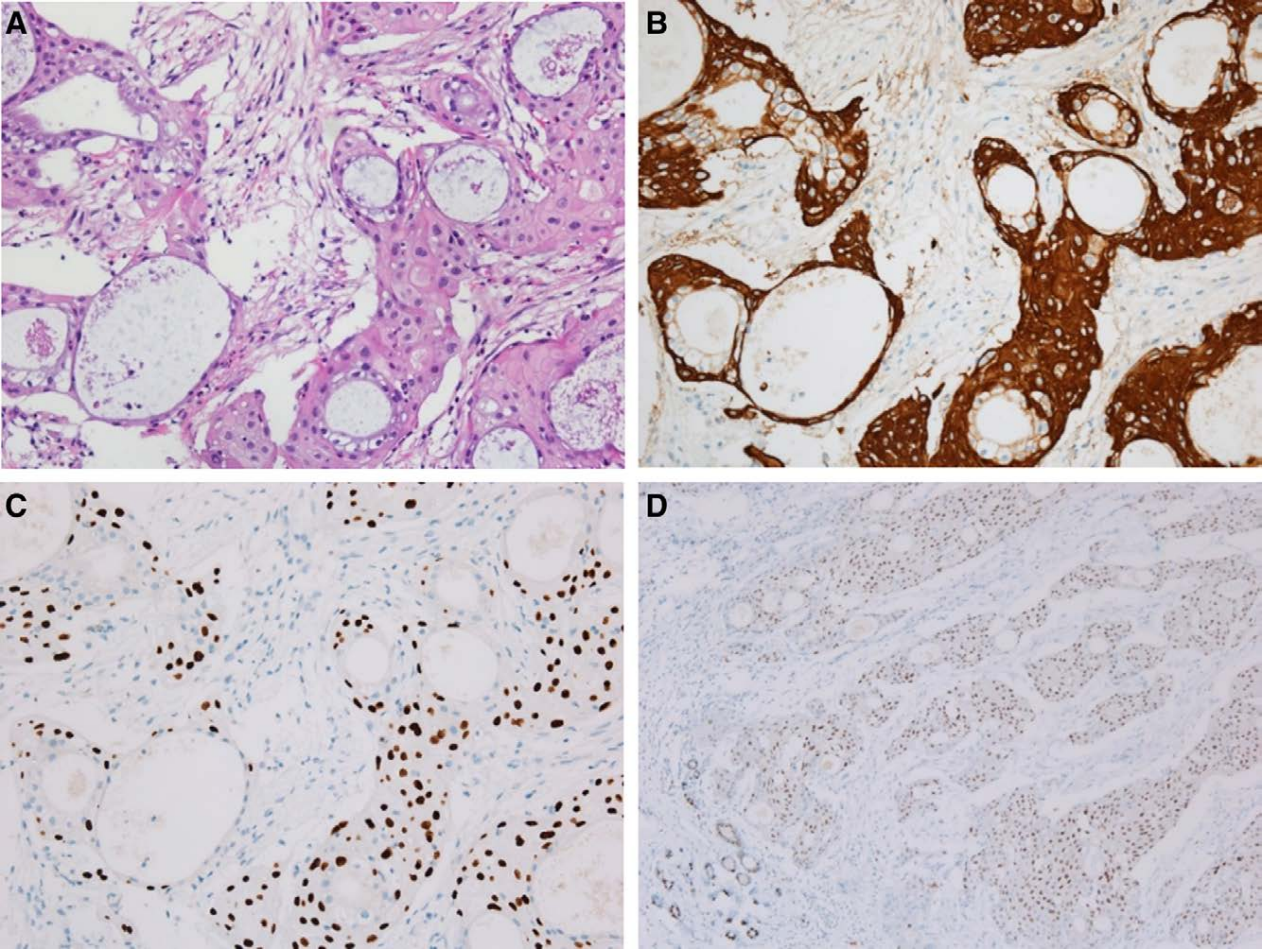
Histopathological examination confirmed MEC of the breast, intermediate grade (A: H&E, ×100, B–D: immunohistochemical stain, B: HMWCK, C: p63, D: ER). (A) The tumor composed of solid tumor cell nests and central cystic area. (B and C) The innermost cell of cystic area is clear mucinous cell negative for HMWCK and p63. The outer surrounding cells are polygonal eosinophilic epidermoid cells positive for HMWCK and p63. Some of the surrounding cells are intermediate cells positive for HMWCK and negative for p63. (D) The tumor cells showing weak expression of the ER. The tumor is positive for Ki-67 and negative for the PR and HER-2 (not shown here). ER = estrogen receptor, H&E = hematoxylin and eosin; HER-2 = human epidermal growth factor receptor 2, HMWCK = high-molecular-weight cytokeratin, MEC = mucoepidermoid carcinoma, PR = progesterone receptor.

The patient underwent breast-conserving surgery with a sentinel lymph node biopsy. Macroscopically, the cut sections of breast-conserving surgery specimens revealed a relatively well-circumscribed, whitish, predominantly solid tumor with scattered tiny cysts measuring 3.2 × 2.5 cm (Fig. 6). On subsequent histopathological examination, the tumor was found to be composed of solid tumor cell nests and a central cystic area (Fig. 7A). The innermost cell of the cystic area was a clear mucinous cell that is negative for high-molecular-weight cytokeratin (HMWCK) and p63. The outer surrounding cells were polygonal eosinophilic epidermoid cells that were positive for HMWCK and p63. Some surrounding cells were intermediate cells that were positive for HMWCK and negative for p63 (Fig. 7B, C). The tumor was confirmed to be an intermediate-grade MEC and was immunohistochemically positive for estrogen receptor (ER; Fig. 7D), p53, and Ki-67 and negative for progesterone receptor and human epidermal growth factor receptor 2. No lymphovascular or neural invasion was identified, and all surgical margins were negative. Sentinel lymph node biopsy revealed no evidence of lymph node metastases. The clinical staging was T2N0M0. Subsequently, the patient underwent adjuvant chemotherapy and radiotherapy followed by hormonal therapy. Over 37 months, she showed no evidence of recurrence on postoperative follow-up examinations.

This study was approved by the Institutional Review Board of the Gyeongsang National University Hospital (Institutional Review Board no. GNUH-2022-04-024), and written informed consent was obtained from the patient.

## 3. Discussion

Although MEC is one of the most common malignancies of the salivary glands,^[1]^ it is an extremely rare entity in other organs, including the breast, thyroid, esophagus, bronchi, pleura, forearm, tonsil, colon, and thymus, with only a few cases being reported.^[3,11]^ Because of its rarity, only 43 cases of breast MEC have been reported in the English literature between 1979 and 2022,^[3,4]^ and the clinicopathological features of breast MEC have not been fully demonstrated.

In an analysis of published cases of breast MEC,^[3]^ the mean patient age was 57 years (standard deviation, 15.2 years), with patient ages ranging from 27 to 86 years. Most patients complained of a palpable lump in their breasts, and very few cases presented with a nipple discharge or no clinical symptoms. The tumor size ranged from 5 to 82 mm, with a median size of 20 mm.

Histopathologically, MEC of the breast shares the same morphological features as those of the salivary glands, which include variable proportions of 4 cell types: basaloid, intermediate, epidermoid, and mucinous cells.^[5,11–15]^ A histopathological diagnosis of breast MEC is based on detection of the 4 cells mentioned previously, and a few tumors such as intraductal papilloma, adenomyoepithelioma, and clear cell hidradenoma may show similar features to breast MEC.^[16]^

According to previous studies, the pathological grade of MEC is an important prognostic factor and the accurate grading is crucial for predicting prognosis.^[6–8,17,18]^ Fujino et al^[7]^ reported that low- and intermediate-grade breast MECs showed a 5-year survival rate of >90%, whereas high-grade MECs showed a 5-year survival rate of 54%.^[17]^ Moreover, Pia-Foschini et al^[18]^ found that patients with low-grade MEC were disease-free in the follow-up period, whereas high-grade MECs usually showed aggressive behavior with metastasis to axillary nodes and distant organs. Histologically, low- and high-grade MECs showed different morphological features.^[8,13]^ Although low-grade MECs may consist of over 50% mucinous cells, possibly forming cystic cavities in the tumor, high-grade MECs may predominantly consist of intermediate and epidermoid cells and less mucinous cells than low-grade MECs. In addition, high-grade MEC may have a tendency to infiltrate tissues around the tumor.^[8]^

Although many reports have described the pathological findings of breast MECs, their radiological features have been rarely investigated, with only a few reports describing the radiological imaging findings for these tumors. We describe a case of intermediate-grade MEC with radiological findings and review the literature focused on the radiological features of breast MECs. Our review of previously published papers showed that only 8 studies provided radiological data and only 2 of these showed imaging data from all 3 modalities: MG, US, and MRI.^[3–10]^ The imaging features of the reported breast MECs are summarized in Table 1. Of the 9 cases with radiological imaging data, 7 involved low-grade MECs and 2 involved intermediate-grade MECs. MG in 6 cases showed a well-defined or ill-defined mass with an oval, lobulated, or irregular shape. Only 1 of these involved coarse calcification in the mass on MG.^[7]^ US examinations in 8 cases showed a solid or complex echoic mass. Five cases of low-grade MEC showed a complex echoic mass with various amounts of anechoic areas representing internal cystic components and 2 cases of intermediate-grade MECs showed a heterogeneous echoic solid mass without internal cystic portion.^[7]^ Only 3 cases involved MRI examinations.^[4,7]^ On MRI, 1 case of low-grade MEC showed an enhancing mass with some areas of high signal intensity on the T2-weighted image, representing cystic components,^[18]^ and 2 cases of intermediate-grade MEC (including our case) showed a heterogeneously enhancing mass without an internal cystic component.^[12]^ Our case is the first to present the tumor enhancement kinetics of MEC, which showed rapid initial enhancement, rapid washout, and kinetic heterogeneity in the tumor. In our case, both US and MRI showed intratumoral heterogeneity in common, with internal echogenicity on US and internal enhancement pattern and kinetics on MRI. Recent studies reported that tumor kinetics and related heterogeneity on dynamic contrast-enhanced MRI are associated with prognosis in women with breast cancer.^[19,20]^ Both morphological and kinetic features of our case on breast MRI are consistent with nonspecific features that breast malignancy usually show on dynamic contrast-enhanced MRI. We found that the radiological findings for breast MEC were variable and not specific enough for diagnosis. Eight of the 9 cases showed masses with suspicious features on radiological examinations (equivalent to BI-RADS category 4), which required tissue confirmation; however, 1 asymptomatic case of low-grade MEC showed a small oval cyst with no initial evidence of malignancy, which was considered a probably benign lesion (equivalent to BI-RADS category 3).^[3]^

**Table 1:** Summary ofpreviously reported cases of breast MEC with radiological findings.

No.	Study (year)	Age, yr	Symptom	Tumor size (cm)	Imaging modalities	Radiological findings	Pathological grade	Hormonal receptor status
1	Horii et al (2006)^[6]^	54	Palpable lump	2.5	MG	A hyperdense and irregular mass with a spiculated margin	NS	ER (+)
					US	An irregular hypoechoic mass without posterior echo enhancement		PR (–)
								HER-2 (1+)
2	Turk et al (2013)^[10]^	40	Palpable lump	4	MG	A large oval hyperdense mass	NS	Triple negative
					US	A regular, solid mass (not shown)		
3	Fujino et al (2016)^[7]^	71	Palpable lump	2	MG	An unclear mass with an accumulation of calcification	Intermediate grade	Triple negative
					US	A hyperechoic lesion within a hypoechoic area, with a rough surface		
					MRI	A heterogeneously enhancing mass		
4	Cheng et al (2017)^[5]^	61	Palpable lump	3	CT	Only a few solid tissue masses within the septa-divided cystic spaces	Low grade	ER (+)
5	Sherwell-Cabello et al (2017)^[9]^	86	Palpable lump	6	US	A predominantly cystic, complex echoic mass	Low grade	Triple negative
6	Ye et al (2020)^[8]^	42	Palpable lump	2.6	US	A predominantly cystic mass	Low grade	Triple negative
7	Metaxa et al (2020)^[3]^	63	No symptom	2.4	MG	A well-defined, lobulated mass	Low grade	ER (weakly +)
					US	A well-defined cystic mass with a thick cyst wall and internal septation		
8	Chen et al (2022)^[4]^	38	Palpable lump	4.2	MG	A well-defined huge mass with a partly lobulated boundary	Low grade	NS
					US	A lobulated and well-defined complex echoic mass		
					MRI	A heterogeneously enhancing mass with a cystic portion		
9	Our case (2022)	47	Palpable lump	3.2	MG	An irregular, microlobulated hyperdense mass	Intermediate	ER (weakly +)
					US	An irregular, circumscribed heterogeneous echoic mass	grade	PR (–), HER-2 (–)
					MRI	An enhancing mass without a prominent cystic portion		

HER-2 = human epidermal growth factor receptor 2, MEC = mucoepidermoid carcinoma, MG = mammography, MRI = magnetic resonance imaging, NS = not stated, PR = progesterone receptor, US = ultrasonography.

We compared the radiologic and pathological findings in the reported cases and found that all cases of low-grade MEC showed cystic components in the mass, and intermediate-grade MECs showed a solid mass without an internal cystic component on US or MRI. This radiologic finding of low-grade MEC is consistent with the pathological finding showing an internal cystic cavity with abundant mucinous cells, unlike intermediate- or high-grade MEC.^[17]^ Our case, in which the tumor was pathologically confirmed as an intermediate-grade MEC, showed no significant cystic components on radiological examinations, consistent with a previously reported case of an intermediate-grade MEC.^[7]^ On the basis of our analyses, the presence of a cystic component in the tumor on radiological examination can be assumed to indicate the pathological grade of breast MEC. Moreover, because of the correlation between pathological grade and prognosis in breast MEC, the presence of cystic components on imaging is likely to be an indicator of a better prognosis. Nevertheless, the number of reported cases with radiological imaging data is very limited, and the imaging features of high-grade MEC have not been reported. Therefore, further studies are needed to discuss the possibility of using imaging findings as predictors of pathological grades and prognosis in breast MEC.

Ye et al^[8]^ reported that breast MEC often presents a triple-negative immunophenotype with the absence of ER, progesterone receptor, and human epidermal growth factor receptor 2.^[21]^ However, according to the study of Pia-Foschini et al,^[18]^ unlike other triple-negative breast cancers, these tumors show a better prognosis. Some studies reported that breast MEC exhibits lower levels of hormonal receptor expression.^[3,5,6]^ Our patient showed weak positivity for ER. Of the 9 cases with radiological imaging data in Table 1, 4 were triple-negative and 4 were ER-positive MEC, with no significant differences in radiological features between triple-negative and ER-positive MECs. The number of reported cases presenting with radiological features is too small to analyze differences in imaging features according to hormonal receptor status. Regarding the relationship between radiological features of MEC and more pathological and biological factors, further research is needed.

The standard therapeutic approach for breast MEC is not well established because of its low incidence. Treatment can include surgery, radiotherapy, chemotherapy, and hormonal therapy based on the tumor size, pathological grade, and nodal status on the preoperative examination, similar to the other types of breast cancers.^[4]^ As mentioned earlier, the prognosis of breast MEC depends on the pathological grade of the tumor and is better for low-grade than for high-grade MEC.

## 4. Conclusion

MEC of the breast is an extremely rare malignancy. Owing to the limited number of previous reports on breast MEC, the clinicopathological and radiological features of this entity have not been fully demonstrated. Radiological imaging showed no specific features for the diagnosis of breast MECs, and the imaging characteristics of MECs are variable, with masses mostly equivalent to BI-RADS category 4. Although the diagnosis of breast MEC depends on pathological examinations, the presence of an internal cystic component on radiological examinations may be an indicator of low-grade tumor and better prognosis. However, further studies are required to draw definitive conclusions.

## Acknowledgment

The authors would like to thank Editage (www.editage.co.kr) for English language editing.

## Author contributions

Conceptualization: Seongjun Bak and Hye Young Choi.

Data curation: Seongjun Bak, Jeong-Hee Lee, and Ju-Yeon Kim.

Supervision: Hye Young Choi.

Validation: Jae Beom Na, Dae Seob Choi, Jae Min Cho, Ho Cheol Choi, Mi Jung Park, and Ji Eun Kim.

Visualization: Jeong-Hee Lee, Hwa Seon Shin, Jung Ho Won, and Jae-Myung Kim.

Writing original draft: Seongjun Bak and Hye Young Choi.

Writing, review, and editing: Hye Young Choi.

## References

[1] Ellis GL Auclair PL eds. Atlas of Tumor Pathology. Tumors of the Salivary Glands. 3rd series, fascicle 17. Washington, DC: Armed Forces Institute of Pathology 1996:155–75.

[2] Fisher ER Palekar AS Gregorio RM. Mucoepidermoid and squamous cell carcinomas of breast with reference to squamous metaplasia and giant cell tumors. Am J Surg Pathol. 1983;7:15–27. 6829846 10.1097/00000478-198301000-00002

[3] Metaxa L Exarchos G Metaxa L. Primary mucoepidermoid carcinoma of the breast: a rare breast entity. Clin Oncol Res. 2020;3:1–9.34142081

[4] Chen G Liu W Liao X. Imaging findings of the primary mucoepidermoid carcinoma of the breast. Clin Case Rep. 2022;10:e05449. 35169478 10.1002/ccr3.5449 PMC8832380

[5] Cheng M Geng C Tang T. Mucoepidermoid carcinoma of the breast: four case reports and review of the literature. Medicine. 2017;96:e938551. 10.1097/MD.0000000000009385 PMC5758243 29390541

[6] Horii R Akiyama F Ikenaga M. Muco-epidermoid carcinoma of the breast. Pathol Int. 2006;56:549–53. 16930336 10.1111/j.1440-1827.2006.02004.x

[7] Fujino M Mori D Akashi M. Mucoepidermoid carcinoma of the breast found during treatment of lymphoma. Case Rep Oncol. 2016;9:806–14. 28101030 10.1159/000452792 PMC5216231

[8] Ye RP Liao YH Xia T. Breast epidermoid carcinoma: a case report and review of literature. Int J Clin Exp Pathol. 2020;13:3192–9. 33425121 PMC7791392

[9] Sherwell-Cabello S Maffuz-Aziz A Ríos-Luna NP. Primary mucoepidermoid carcinoma of the breast. Breast J. 2017;23:753–5. 28397345 10.1111/tbj.12819

[10] Turk E Karagulle E Erinanc OH. Mucoepidermoid carcinoma of the breast. Breast J. 2013;19:206–8. 23294278 10.1111/tbj.12080

[11] Hornychová H Ryska A Betlach J. Mucoepidermoid carcinoma of the breast. Neoplasma. 2007;54:168–72. 17319792

[12] Di Tommaso L Foschini MP Ragazzini T. Mucoepidermoid carcinoma of the breast. Virchows Arch. 2004;444:13–9. 14634807 10.1007/s00428-003-0923-y

[13] Cameselle-Teijeiro J Febles-Pérez C Sobrinho-Simões M. Papillary and mucoepidermoid carcinoma of the thyroid with anaplastic transformation: a case report with histologic and immunohistochemical findings that support a provocative histogenetic hypothesis. Pathol Res Pract. 1995;191:1214–21. 8927569 10.1016/S0344-0338(11)81129-5

[14] Choi D Kim H Lee KS. Mucoepidermoid carcinoma of the liver diagnosed as a liver abscess: report of a case. Surg Today. 2004;34:968–72. 15526136 10.1007/s00595-004-2820-7

[15] Hanna W Kahn HJ. Ultrastructural and immunohistochemical characteristics of mucoepidermoid carcinoma of the breast. Hum Pathol. 1985;16:941–6. 4029947 10.1016/s0046-8177(85)80133-7

[16] Memon RA Prieto Granada CN Wei S. Clear cell papillary neoplasm of the breast with MAML2 gene rearrangement: clear cell hidradenoma or low-grade mucoepidermoid carcinoma? Pathol Res Pract. 2020;216:153140. 32853960 10.1016/j.prp.2020.153140

[17] Yan M Gilmore H Harbhajanka A. Mucoepidermoid carcinoma of the breast with MAML2 rearrangement: a case report and literature review. Int J Surg Pathol. 2020;28:787–92. 32362174 10.1177/1066896920916779

[18] Pia-Foschini MP Reis-Filho JS Eusebi V. Salivary gland-like tumors of the breast: surgical and molecular pathology. J Clin Pathol. 2003;56:497–506. 12835294 10.1136/jcp.56.7.497 PMC1769991

[19] Kim JY Kim JJ Lee H. Kinetic heterogeneity of breast cancer determined using computer-aided diagnosis of preoperative MRI scans: relationship to distant metastasis-free survival. Radiology. 2020;295:517–26. 32228293 10.1148/radiol.2020192039

[20] Nam SY Ko ES Lim Y. Preoperative dynamic breast magnetic resonance imaging kinetic features using computer-aided diagnosis: association with survival out-come and tumor aggressiveness in patients with invasive breast cancer. PLoS One. 2018;13:e0195756. 29649266 10.1371/journal.pone.0195756 PMC5896992

[21] Jones C Ford E Gillett C. Molecular cytogenetic identification of subgroups of grade III invasive ductal breast carcinomas with different clinical outcomes. Clin Cancer Res. 2004;10:5988–97. 15447982 10.1158/1078-0432.CCR-03-0731

